# Psychometric properties of the OLQ-13 scale to measure Sense of Coherence in a community-dwelling older population

**DOI:** 10.1186/1477-7525-9-37

**Published:** 2011-05-23

**Authors:** Jenneken Naaldenberg, Hilde Tobi, Franciska van den Esker, Lenneke Vaandrager

**Affiliations:** 1 Health and Society group, Wageningen University, Hollandseweg 1 Wageningen, The Netherlands; 2 Research Methodologies Group, Wageningen University, Hollandseweg 1 Wageningen. The Netherlands

## Abstract

**Background:**

With the ongoing demographic shift, the quality of life and health promotion among older individuals are becoming increasingly important. Recent research suggests that Sense of Coherence positively affects quality of life. Hence, a valid and reliable measurement of Sense of Coherence is pivotal. The 13-item Orientation to Life Questionnaire (OLQ-13) can be used to measure Sense of Coherence. The purpose of the present study is to assess the psychometric properties, validity, and reliability, of the OLQ-13 in community-dwelling individuals, aged 65 and older.

**Methods:**

The OLQ-13 scale was administered as part of a healthy aging project for non-institutionalized people aged 65 years and older. Internal consistency and reliability were assessed by means of inter-item and test-halves correlations and Cronbach's alpha. Construct validity was explored using cluster analysis and exploratory factor analysis (n = 703) and tested using confirmatory factor analysis on a separate subset of individuals (n = 658). Item face validity was investigated by means of 12 semi-structured interviews.

**Results:**

The reliability and the validity of the OLQ-13 in this population of non-institutionalized individuals aged 65 years and older was ambiguous, at least partly due to the poor performance of two items (b and d), which was confirmed by results from the qualitative part of this study. The psychometric properties of the proposed OLQ-11, obtained by deleting the two items, were better. In particular, the interpretation of exploratory factor solution improved. Whereas the underlying theoretical constructs could not be linked to the exploratory analyses of OLQ-13, this was to some extent possible in OLQ-11. The superior validity of OLQ-11 over OLQ-13 was supported by the better model fit in the confirmatory factor analysis.

**Conclusions:**

The present mixed-method study suggests the proposed OLQ-11 as a more suitable instrument for measuring Sense of Coherence than the OLQ-13 in a population of ageing individuals. This study confirms that the validity and reliability of OLQ-13 may differ substantially in different populations.

## Background

Salutogenesis offers a theoretical approach to health promotion in which Sense of Coherence (SOC), the ability to use available resources in a health promoting way, takes a central place [1-3]. Within the salutogenic theory, Sense of Coherence is described as a global orientation that expresses the extent to which individuals have a feeling of confidence that their environment is structured, predictable and explicable; resources are available to meet challenges; and these challenges are worth engaging in [1]. Following this description, Sense of Coherence is further conceptualized by three different dimensions: a) comprehensibility, the cognitive component, b) manageability, the instrumental component and c) meaningfulness, the emotional component [2,4,5]. Together, these components reflect the interactions of an individual with resources in the environment. Individuals with a high SOC are expected to be confident that they have control over their situation and know how to act in a health promoting way [4,6].

Sense of Coherence in individuals is usually measured by the 29-item orientation to life questionnaire (OLQ) or the shorter 13-item version of this questionnaire [2,7]. Items in these questionnaires are designed to measure one of the SOC dimensions: meaningfulness (ME, 8 resp. 4 items), manageability (MA, 10 resp. 4 items) and comprehensibility (CO, 11 resp. 5 items). Items are scored on 7-point scales. Missing values are not allowed in computing a sum score for an individual. Scores for each sub dimension may be computed as well [2].

The OLQ has been translated and used in many countries and in different populations [7]. A general problem concerning the OLQs is that the three conceptual dimensions never appear clearly from the data in factor analyses [2,4]. Several studies have investigated the factor structure of the OLQs by both exploratory and confirmatory techniques. Some of these studies suggested a one factor structure [8-10], whereas others proposed several factors, not necessarily in line with the original dimensions [11,12]. Studies using confirmatory techniques presented a better fit for models with the three dimensions related to SOC (second order three factor models) than models relating the individual items to SOC (one factor models) [10,13-15]. Overall, the factorial structure of OLQ seems to be multi-dimensional rather than one dimensional [7].

Recent research suggests that interventions aimed at aging populations can benefit by taking into account concepts like Sense of Coherence of individuals in the target population [16-21]. Hence, a valid and reliable measurement of Sense of Coherence is pivotal. The aim of this study is to assess the psychometric properties, validity and reliability of the OLQ-13 in community-dwelling older population, aged 65 and over.

## Methods

### Data and study population

This study used data from a large healthy ageing project [22] for which a random study sample of 4,050 non-institutionalized people aged 65 years and over was selected from the municipal registration system of three participating municipalities in the eastern part of the Netherlands. Because the response rate in the oldest age group 75 year and over was expected to be smaller than the age group 65 to 74 years, this group was oversampled to constitute half of the study population. Data were collected in August 2008 by means of a 60-item, self-administered questionnaire that included the existing Dutch OLQ-13 [23]. Since this study was not invasive to the participant's integrity, it did not require a formal ethics review following the criteria of the Medical Research Involving Human Subjects Act. The use of personal data in this study was in compliance with the Dutch Personal Data Protection Act and the Municipal Database Act. It has been registered with the Dutch Data Protection Authority under number1440826 [22].The response rate to this survey was 67%. To limit multiple use of the same data for future analyses, a random subset of 1,361 respondents was used in this study.

Five items in the OLQ-13 questionnaire were reverse-coded in order to score in the right direction (high score meaning high SOC, see table 1). Missing data were handled as appropriate to each specific analysis being pairwise deletion for correlations, list wise exclusion for exploratory factor analyses and full estimation maximum likelihood estimation in the confirmatory factor analyses. Where the focus was on the items within the scale (cluster analyses, Cronbach's alpha) all respondents on these items were included.

**Table 1:** Overview items characteristics and test halves composition (N = 1361)

Item (dim)	Question							Mean Sd	th 1 a/b	th 2 a/b	th 3 a/b
A (ME)	Do you have the feeling that you don't really care about what goes on around you? (recode)							5.38	a	b	b
	1	2	3	4	5	6	7	1.49			
	Seldom or never				Very often						

B (CO)	Have you ever been surprised by the behavior of people you thought you knew well? (recode)							4.69	a	b	b
								1.77			
	1	2	3	4	5	6	7				
	Never happened				Always happened						

C (MA)	Have people you counted on, disappointed you? (recode)							4.80	a	a	a
	1	2	3	4	5	6	7	1.78			
	Never happened				Always happened						

D (ME)	Until now your life has had:							4.64	a	a	a
	1	2	3	4	5	6	7	1.72			
	No clear goals or purposes at all				Very clear goals or purposes						

E (MA)	Do you have the feeling that you are being treated unfairly?							5.41	a	a	a
	1	2	3	4	5	6	7	1.60			
	Very often				Seldom or never						

F(CO)	Do you have the feeling that you are in an unfamiliar situation and don't know what to do?							5.69	a	a	b
	1	2	3	4	5	6	7	1.44			
	Very often				Seldom or never						

G (ME)	The things you do every day are (recode):							5.25	a	b	a
	1	2	3	4	5	6	7	1.20			
	A source of deep pleasure and satisfaction				a source of pain and boredom						

H (CO)	How often are you're feelings and ideas mixed-up?							5.82	b	a	a
	1	1.40	2	3	4	5	6	7			
	Very often				Seldom or never						

I (CO)	Do you sometimes have feelings you would rather not have?							5.31	b	b	b
	1	2	3	4	5	6	7	1.55			
	Very often				Seldom or never						

J (MA)	Many people - even those with a strong character - sometimes feel unlucky in certain situations. How often have you felt this way in the past? (recode)							5.21 1.60	b	b	b
	1	2	3	4	5	6	7				
	Seldom or never				Very often						

K (CO)	When something happened, do you in your opinion usually:							4.97	b	a	b
	1	1.36	2	3	4	5	6	7			
	Over- or underestimate it's importance ... saw things in the right proportion.										

L (ME)	How often do you have the feeling that there's little meaning in the things you do in your daily life?							5.49 1.38	b	a	a
	1	2	3	4	5	6	7				
	Very often				Seldom or never						

M (MA)	How often do you have feelings of which you're not sure if you can control them?							5.56 1.41	b	b	a
	1	2	3	4	5	6	7				
	Very often				Seldom or never						

### Reliability and internal consistency

Inter-item correlations, split-half correlations and Cronbach's alpha were used to investigate reliability and internal consistency. Items were correlated using a standard Pearson's correlation. The split-half reliability procedure was repeated three times, each using different ways to split OLQ-13 into two halves, after which the Spearman-Brown split-half reliability coefficient was computed. The first approach followed a standard procedure for split-half analysis in which the OLQ-13 was split into halves based on order of items (resulting in a test half consisting of items a to g and a test half with items h to m). Next, to create more equivalent halves, different dimensions of Sense of Coherence were taken into account and divided equally over the test halves. In the third approach, the OLQ-13 was divided into test halves based on item score means and standard deviation. Table 1 provides an overview of items, questions, and test halves. Cronbach's alpha was computed for the OLQ-13 as a whole as well as for each Sense of Coherence sub dimension.

### Validity

In order to investigate construct validity, the data were randomly divided over a construction set of size n = 703 and a confirmation set of size n = 658. First, the construction set was used to explore whether the theoretical dimensions meaningfulness, manageability and comprehensibility would appear within the data.

Structures within the data were explored by means of exploratory factor analysis using principal axis factoring with oblique rotation. Screeplots, Eigenvalues > 1, and Horn's parallel procedure [24] were used to assess the number of factors to extract. Also, a hierarchical cluster analysis applying Ward's method was used to identify homogenous groups of variables. Ward's method minimizes distances within groups while maximizing differences between group and thus provides the best chance of identifying relevant clusters of items [25].

Secondly, face validity of the items was investigated based on 12 (six male, six female) face-to-face semi-structured interviews. Interviewees were living within the same geographical district as used for the survey. Six interviewees were aged 65 to 74 years, the others were 75 or older. Interviewees were asked to complete the OLQ-13 (Dutch translation as used in the survey) and were probed to elaborate on the meaning and relevance of items and their interpretation of the questions and answering scales. Transcripts were made verbatim. Data were analyzed for content in the response per item and the cognitive process of scoring. Firstly, a domain analysis was performed for the response per item and the cognitive process of scoring in order to obtain emic accounts of the interviewees [26]. This was followed by a content analysis, in order to describe aspects of Sense of Coherence as expressed by the interviewees [27]. The discussion of results will focus on those items that are of interest to the overall aim of this paper.

Finally, results from both the explorative analyses and the qualitative analysis were used in the confirmative stage to test different structures using the confirmation dataset. Confirmatory factor analyses with full information maximum likelihood estimation (FIML) was used to estimate the models. FIML estimation yields consistent and efficient estimates when data are missing at random [28]. Fit indices Chi^2^, CFI and RMSEA were compared for each of the fitted models.

Analyses were performed using PASW 17 software, and AMOS 17 software for the confirmatory factor analyses (http://www.spss.com). The analysis of the qualitative data was supported by the use of Atlas ti. software (http://www.atlasti.com/).

## Results

### Descriptives

Of the 1,361 included respondents, 43% were men and 57% women, with ages ranging from 65 to 101 (Mean = 75, SD = 6.8) and 49.4% in the age group 65-74. Table 2 summarizes the descriptives for the OLQ-13 questionnaire in the present sample.

**Table 2:** Descriptives for the Orientation to life questionnaire, OLQ-13 (n = 1361)

	Orientation to life questionnaire (13 items)	Meaningfulness (4 items)	Manageability (4 items)	Comprehensibility (5 items)
**Mean Sum score**	68	20	20	26

**Sd**	10	3.8	4.3	4.8

**Missing**	248	177	151	200

### Internal consistency and reliability

#### Inter-item correlations

Inter-item correlations are provided in table 3. All correlations were positive except for the negative correlations of item d with item b, and item d with item c after recoding of those items. The correlation between item c and b is remarkably high at r = .718.

**Table 3:** Inter-item correlations orientation to life questionnaire, OLQ-13 (n = 1361)

	A	B	C	D	E	F	G	H	I	J	K	L	M
**A**	1	-	-	-	-	-	-	-	-	-	-	-	-
**B**	.189	1	-	-	-	-	-	-	-	-	-	-	-
**C**	.184	.718	1	-	-	-	-	-	-	-	-	-	-
**D**	.089	-.112	.-.079	1	-	-	-	-	-	-	-	-	-
**E**	.132	.176	.223	.154	1	-	-	-	-	-	-	-	-
**F**	.138	.109	.191	.224	.328	1	-	-	-	-	-	-	-
**G**	.219	.185	.209	.181	.157	.277	1	-	-	-	-	-	-
**H**	.151	.087	.121	.171	.278	.471	.277	1	-	-	-	-	-
**I**	.084	.152	.209	.148	.257	.403	.326	.572	1	-	-	-	-
**J**	.122	.263	.327	.083	.254	.248	.310	.271	.323	1	-	-	-
**K**	.132	.081	.143	.212	.200	.304	.112	.311	.262	.101	1	-	-
**L**	.242	.129	.181	.252	.238	.352	.392	.378	.390	.227	.263	1	-
**M**	.201	.169	.180	.176	.266	.442	.289	.527	.519	.288	.322	.436	1

#### Split-half reliability

The correlation coefficient for halves 1a/b (see table 1) was r = .60 with a reliability coefficient of .75. The equal division of theoretical dimensions over test halves (2a/b, table 1) gave a correlation coefficient of r = .68 and a reliability coefficient of .80. When halves were composed data driven (3a/b, table 1), an r = .76 and a reliability coefficient of .86 were obtained.

#### Cronbach's alpha

The Cronbach's alpha for the OLQ-13 was .80. The dimensions scored lower, with the alpha for meaningfulness at .53; the alpha for manageability at .58; and the alpha for comprehensibility at .64. Deleting items resulted in marginal improvements, deleting item b from the comprehensibility dimension increased the alpha to .71.

### Validity

The high correlation of item b with item c, the improved Cronbach's alpha on deletion of item b and the negative correlations of item d with both items, gave cause to delete both item b and item d, thus obtaining OLQ-11. Validity analyses were therefore performed on both the OLQ-13 and the OLQ-11.

#### Exploratory factor analysis

In both the OLQ-13 and the OLQ-11, there was no unambiguous indication of the number of factors to extract. Examination of the screeplot indicated a one factor solution, whereas the Eigenvalues > 1 criterion and Horn's parallel procedure suggested a three factor solution. The pattern matrix for the three factor solution in both the OLQ-13 and the OLQ-11 is presented in table 4. The factor solution for the OLQ-13 was hard to interpret. The solution for the OLQ-11 seemed less ambiguous with regard to the theoretical dimensions of Sense of Coherence. Factor correlations between the three factors ranged from r = 1.8 to r = .52 in the OLQ-13, and r = .44 to r = .45 in the OLQ-11.

**Table 4:** Pattern matrix principal axis factoring, oblique rotation (n = 703)

		Factor solution OLQ-13			Factor solution OLQ-11		
item	dim	1	2	3	1	2	3
A	Me	-	-	.408	-	-	.405
B	Co	-	.753	-			
C	Ma	-	.880	-	-.150	.669	-
D	Me	.128	-.223	.348			
E	Ma	.285	.198	.102	.160	.368	-
F	Co	.512	-	.163	.455	.165	.120
G	Me	.142	-	.409	.117	.126	.374
H	Co	.818	-	-	.850	-	-
I	Co	.787	-	-	.714	.101	-
J	Ma	.363	.299	-	.139	.576	
K	Co	.259	-	.327	.283	-	.266
L	Me	.109	-	.699	.167	-	.728
M	Ma	.627	-	.182	.624	-	.206
Explained variance		32% one factor/42% three factor			35% one factor/40% three factor		

Values below .100 suppressed

#### Cluster analysis

**Figure 1:**
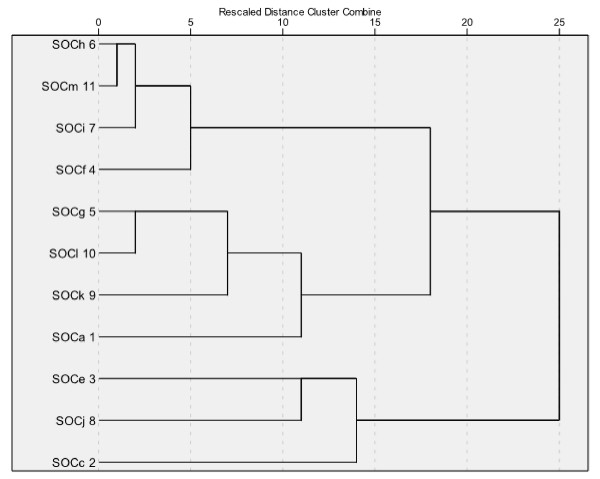
**Dendrogram cluster analysis without item b & d (N = 703)**.

Cluster analysis of the OLQ-13 resulted in a hard-to-interpret and messy structure in which the theoretical Sense of Coherence dimensions were not identifiable. Again, the OLQ-11 showed a more coherent picture with more resemblance to the theoretical dimensions. However, the strongest cluster was found between two items designed to represent manageability and comprehensibility. This is illustrated by the dendrogram in figure 1.

#### Item face validity

Overall, interviewees encountered difficulties in answering and interpreting at least some of the questions in the OLQ-13. More specifically, item b) " Have you ever been surprised by the behavior of people you thought you knew well?" and item c) "Have people you counted on, disappointed you?" were frequently interpreted as addressing the same issue. Interviewees were missing the nuance, and the phrasing "surprised" in the question for item b was perceived in a negative connotation, as a disappointment. When probed, interviewees perceived item c as relating to the manageability dimension. Item b, however, was not perceived by the interviewees as relating to any of the theoretical dimensions.

Item d) "Until now your life has had: No clear goals or purposes at all ... Very clear goals or purposes" was perceived as referring to the future. Interviewees related these future goals to the context of occupation and work, and therefore did not think it was applicable to aging individuals who had already retired. When probed, interviewees related this item to the meaningfulness dimension.

With regard to item k) "When something happened, do you in your opinion usually: Over- or underestimated it's importance ... saw things in the right proportion" interviewees struggled to answer the question. This item was regarded as difficult, unclear and too broad. Interviewees had no clear view about the dimension to which this item relates.

Item m) "How often do you have feelings of which you're not sure if you can control them?" was perceived as relating to negative emotions. Interviewees mentioned: anger, loneliness, tensions and reluctance and the degree of control over these emotions. This item was perceived to relate to the comprehensibility dimension.

### Confirmatory factor analysis

Confirmatory factor analysis was performed on both OLQ-13 and OLQ-11 using the confirmation set. A one factor model connecting the items directly to one factor being Sense of Coherence was tested, followed by a first order model in which the three dimensions were included. The fit indices for the tested models: Chi^2^, RMSEA and GFI, as well as the factor Sense of Coherence and dimension variances, are provided in table 5. The Chi^2 ^values in all models were highly significant due to over sensitivity to the large sample size. The unique factor variances in all models are up to ten times larger than the factor Sense of Coherence or the dimension's variances, respectively. The OLQ-13 first order model failed to run properly because the covariance matrix was not positive definite, and a Chi^2 ^could not be computed.

**Table 5:** CFA fit indices and variance (n = 658)

	*Df*	*Chi^*	*CFI*	*RMSEA*	*Var SOC*			*Range error var.*
OLQ-13 one factor model	65	618	.731	.114	.22			1.01 to 3.00
OLQ-11 one factor model	44	142	.930	.058	.21			.99 to 2.96
					***Var. me***	***Var. ma***	***Var. co***	
OLQ-13 first order model	na	na	.723	.115	.28	.48	.75	.87 to 2.92
OLQ-11 first order model	41	123	.941	.055	.28	.41	.77	.88 to 2.95

For both the OLQ-13 and the OLQ-11, the second order model construction resulted in a model that was mathematically equivalent to the one factor models and therefore is not included in the table.

**Figure 2:**
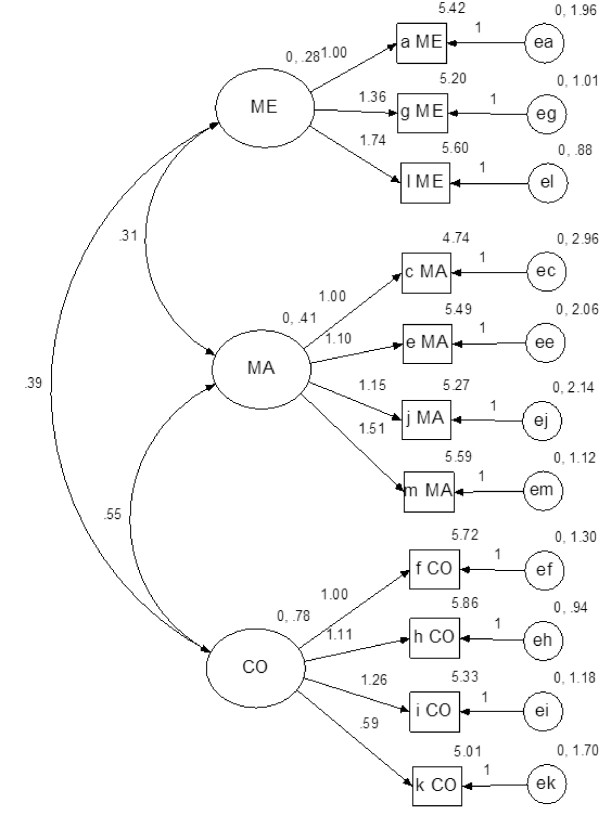
**Path diagram 11 item first order CFA model (N = 658)**.

Compared to the one factor Sense of Coherence model, the first order model yielded better fit indices. Overall, the OLQ-11 models performed better than the OLQ-13 models. The path diagram and parameters for the best fitting first order OLQ-11 model is provided in figure 2.

## Discussion

The reliability and the validity of the OLQ-13 in this population of non-institutionalized individuals aged 65 years and older was ambiguous, at least partly due to the poor performance of item b and item d. This problem has been reported before and seems to be resolved, at least partly, by excluding the items b and d from the analysis [29]. The psychometric properties of the OLQ-11 (obtained by deleting the two items) were indeed better, in particular the exploratory factor solution. Where the underlying theoretical constructs could not be linked to the exploratory analyses of OLQ-13, this was to some extent possible in OLQ-11 with problems remaining due to items k and item m. The superior validity of OLQ-11 over OLQ-13 was supported by the better model fit in the confirmatory factor analysis. It is important, however, to acknowledge the relatively large unique factor variances in the model, suggesting substantial "noise".

The reported high correlation between item b and item c has also been found in other studies [10,29,30], and could be explained in the present study by interviewees perceiving these questions as similar.

The present study confirmed the multi-dimensional nature of SOC in the aging population, previously shown for other populations [8-10,31]. Whether to view Sense of Coherence as a one factor model or a three factor model has often been debated in the literature [2,8-10,15,32,33]. This study therefore applied several criteria to extracting factors in the exploratory analyses, eventually suggesting a three factor model. Moreover, both the three factor and one factor solution were further tested in confirmatory analyses.

The present study focused on the reliability and the construct and face validity of the OLQ-13 among community-dwelling older individuals and suggested the OLQ-11. It did not look into the divergent validity by investigating whether SOC appears as a salutogenic construct distinct from quality of life and (absence of) depression. That this may be an issue was shown in a study on girls aged 14 to 18 years [34]. Blom et al [34] concluded that symptoms of anxiety and depression were better captured in the OLQ-29 than in the specialized scales they considered. It would be interesting to investigate whether similar issues arise in clinical and non-clinical groups of older individuals.

The OLQ-13 has been translated and used in many countries and in different populations. Generally speaking, translation of scales and questionnaires requires explicit attention since translation may influence validity [35]. No published information is available on the translation difficulties and resulting (lack of) equivalence of the OLQ-13 in Dutch.

Although a response rate of 67% is generally considered sufficient, this does not imply full representativeness of the population under study per se. In this study the percentages for the men and women match those of the general population (42% men and 57% women) for the participating municipalities [36]. The over-sampling of the age group 75 and over prevented the bias of under representation of this group. However, this study aimed to assess the OLQ-13 scale and the items within it rather than to draw conclusions on the population under study. This makes issues of representativeness of less importance.

This study is unique in its mixed-method approach to the study of OLQ: it combines of qualitative and quantitative approaches/data. The added value of this combination lies in the quantitative part providing enough data to work with a separate construction and validation set, and the qualitative part of this study providing the detailed in-depth information necessary to understand what made particular items problematic for members of this population. The qualitative part gave the context that allowed for a better understanding of problems that presented themselves in individual items. Since the data for the qualitative part were collected before the quantitative analyses, they can be regarded as independent: neither the quantitative nor the qualitative data collection was influenced by previous results.

Interpretation of the Cronbach's alpha scores is problematic since these alpha scores are influenced by the number of items tested, which were higher for the scale as a whole and the comprehensibility dimension. This problem is illustrated by Olsson [37] in a study comparing the OLQ-29 and OLQ13, showing higher values for the OLQ-29. This study therefore also used split-half and inter-item correlations to assess reliability.

## Conclusion

The present mixed-method study suggests the proposed OLQ-11 as more suitable than the OLQ-13 in this population of aging individuals. In addition, this study illustrates that the validity and reliability of OLQ-13 may differ substantially for different populations since it was shown that aging individuals did not always interpret the questions as intended. Further qualitative investigation of interpretations by other populations is therefore advisable.

## Competing interests

The authors declare that they have no competing interests.

## Authors' contributions

JN and HT carried out the statistical analyses and drafted the manuscript. FE performed and analyzed the qualitative part of this study. LV helped to draft the manuscript and contributed to the analyses of the qualitative data. All authors read and approved the final manuscript.
